# Ultrafast charge transfer enhanced nonlinear optical properties of CH_3_NH_3_PbBr_3_ perovskite quantum dots grown from graphene

**DOI:** 10.1515/nanoph-2022-0251

**Published:** 2022-05-27

**Authors:** Ye Yuan, Fenglin Cao, Peng Li, Jiawen Wu, Baohua Zhu, Yuzong Gu

**Affiliations:** Physics Research Center for Two-Dimensional Optoelectronic Materials and Devices, School of Physics and Electronics, Henan University, Kaifeng 475004, China

**Keywords:** carrier dynamics, CH_3_NH_3_PbBr_3_-G composites, nonlinear optical property, ultrafast charge transfer

## Abstract

Halide perovskite quantum dots (PQDs) have exhibited significantly superior nonlinear optical properties compared to traditional semiconductor materials thanks to their peculiar physical and electronic structures. By further improving the nonlinear optical properties of PQDs, it is expected to adapt to ultrafast photonics applications. This work reported the nonlinear optical properties of methylammonium lead bromide-graphene (CH_3_NH_3_PbBr_3_-G) composites synthesized by growing CH_3_NH_3_PbBr_3_ quantum dots directly from a graphene oxide lattice. Our experiments indicate that the combined advantages of the ultrafast charge transport properties from graphene and the strong charge generation efficiency of perovskite can be integrated together. The CH_3_NH_3_PbBr_3_-G composite exhibited enhanced saturable absorption properties with large modulation depth and very low saturation intensity. The transient absorption spectra and carrier dynamics analysis revealed that the enhancement of the saturated absorption properties of the composites mainly arose from the ultrafast charge transfer between G and CH_3_NH_3_PbBr_3_ which promoted the coupling between different states. The results pave the way for the design of optical switches or mode lockers based on saturable absorbers with good performance.

## 1 Introduction

Halide perovskites have been widely studied in recent years due to their promising applications in the field of photonics [1, 2]. Because of their novel optical and electrical properties including long photocarrier lifetime, high fluorescence yield and tunable absorption and photoluminescence [3–6], perovskite have become star materials and have been used in various optoelectronic devices such as photodiodes, solar cells and photodetectors [7–12]. Recent research shows that halide perovskites also exhibited excellent properties in nonlinear optics. Pal et al. reported that the nonlinear optical (NLO) behaviour of organometal halide perovskites switched over from saturable absorption (SA) to reverse saturable absorption under femtosecond laser pulse excitation, which is attributed to the interplay between single and two-photon absorption by the carriers in the conduction band [13]. Perovskites with different structures have also been investigated and found to have improved NLO properties, such as 2D perovskites [14], chiral perovskites [15] and PQDs [2]. The PQDs may show improved nonlinear absorption properties due to their strong quantum confinement and exciton resonance. Li et al. demonstrated the excellent two-photon absorption (TPA) of CsPb(Br/I)_3_ quantum dots by using open Z-scan techniques. The TPA of perovskites was several times higher than that of typical metal chalcogenide quantum dots such as CdTe/CdS and CdSe/CdZnS [16]. Lu et al. compared the results of Z-scan of CsPbBr_3_ and MAPbBr_3_ QDs with the 800 nm laser and proposed that different structures lead to SA in the former and TPA in the latter [17]. The excellent nonlinear absorption properties demonstrate that PQDs can be developed into some unique devices, such as Q switches, upconversion lasers and infrared detectors [18].

Although perovskites have displayed good performance in NLO, their optical nonlinearity needs to be further improved to meet device requirement. The recent studies were mainly focused on the improvement of NLO properties by regulating the size or structure of perovskites [19–21]. The studies of the effect of the composites on the NLO properties of perovskites have not yet explored in depth. Graphene (G) has been regarded as the excellent material for electronic and optoelectronic applications due to its high carrier mobility and broadband absorption and remarkable stability [22, 23]. In addition, graphene also could be an excellent substrate for growing other semiconductor nanocrystals owing to its rich chemical groups and large surface area to volume ratio. Various graphene-based composites, such as G/Cu_2_Se [24], G/MoS_2_ [25] and G/CdS [26], have shown enhanced nonlinear optical properties, in which the charge transfer process plays an important role. The construction of PQDs-G composite structure will hopefully combine the advantages of both to obtain nonlinear optical materials with excellent performance.

The organic-inorganic hybrid perovskites feature bulky organic sites, which provide possibilities to introduce composite structure [27]. And the CH_3_NH_3_PbBr_3_ QDs have suitable bandgap which can achieve enhanced nonlinear absorption in the resonant absorption region. Therefore, we chose CH_3_NH_3_PbBr_3_ QDs as PQDs to synthesize PQDs-G composite structure in this work. The interaction between graphene and perovskite provides a possible channel for charge transfer, which is expected to improve the nonlinear absorption of the composite. Herein, we have studied the NLO properties through the picosecond Z-scan technology and investigated the transient absorption spectra and the carrier dynamics of CH_3_NH_3_PbBr_3_-G composites by pump-probe technology. Our study indicates that the ultrafast charge transfer between graphene and perovskite effectively improved the nonlinear absorption and the CH_3_NH_3_PbBr_3_-G composites could potentially be used as a passive Q-switcher and mode-locker in ultrafast lasers.

## 2 Experimental

### 2.1 Synthesis of CH_3_NH_3_PbBr_3_-G

Graphene oxide (GO) was synthesized by modified Hummers method [28]. A mixture of graphite powder and potassium permanganate was slowly added to the solution of 10 mL phosphoric acid and 90 mL sulphuric acid. The mixed solution was heated to 50 °C and stirred for 24 h. Next the reactant and hydrogen peroxide solution were mixed with ice bath ultrasound. The reaction products were then purified by washing several times with hydrochloric acid and deionized water. Finally, the GO products were obtained via freeze-drying.

We initiate the growth of CH_3_NH_3_PbBr_3_ QDs directly on the active sites of GO surfaces. Firstly, the obtained GO was dissolved in toluene and sonicated for 2 h to form solution. Then 0.6 mL oleic acid and 120 μL n-octylamine were added to the prepared solution and marked as solution A, 0.2 mmol PbBr_2_ was dissolved in 0.3 mL N,N-dimethylformamide (DMF) to form solution B, 0.22 mmol CH_3_NH_3_Br was dissolved in 0.5 mL DMF to form solution C. Next, the solution B and C were added into solution A in an oil bath at 180 °C. Finally, 8 mL of acetonitrile was added to the reaction solution to initiate the nucleation of CH_3_NH_3_PbBr_3_ QDs. The product was with ethyl acetate and washed with hexane. In this study, CH_3_NH_3_PbBr_3_ and GO with mass ratios of 5:1, 5:3 and 1:1 were obtained by changing the dosage of GO, which we marked as PQDs-G1, G-PQDs-G3 and PQDs-G5, respectively. Pure CH_3_NH_3_PbBr_3_ QDs were synthesized by the same process which we marked as PQDs.

### 2.2 Characterization

Transmission electron microscopy (TEM) images were performed with an electron microscope JEM-2100 (JEOL Ltd. Inc., Akishima, Tokyo, Japan), operating at an acceleration voltage of 100 kV. Energy dispersive X-ray spectroscopy (EDS) was recorded at a voltage of 15 kV by JEOL JSM-7610F. X-ray diffraction (XRD) was performed on Bruker D8Advance (XRD, Bruker D8 Advance, Bruker Inc., Karlsruhe, Badensko-Wuertembersko, Germany). Raman spectra were observed on a Renishaw RM-1000 laser Raman microscope system. The X-ray photoelectron spectroscopy (XPS) spectra were measured with Thermo ESCALAB 250XI (Thermo Fisher Scientific Inc., USA). UV–Vis absorption spectra were obtained with a CARY5000 spectrometer (Agilent Corporation, Sacramento, California, USA). The steady-state photoluminescence (PL) spectra were recorded on a PerkinElmer LS 55 luminescence spectrometer. The time-resolved photoluminescence (TRPL) spectra were measured by fluorescence lifetime spectrometer (FLS980E-S1S1-tm, Edinburgh Instruments).

The third-order nonlinear optical properties of the samples were investigated by Z-scan technique. In the Z-scan system, a Nd:YAG pulse laser (EKSPLA, PL2251) was used with the wavelength of 532 nm, the pulse width of 30 ps and the pulse repetition of 10 Hz. The laser was focused on the sample through a lens with a focal length of 250 mm, and the beam waist radius was 10.6 μm. The femtosecond transient absorption measurements were performed by using a pump-probe setup, consisting of a regenerative amplified Ti:sapphire laser system (Astrella Vitara-S, Coherent) with the pulse width of 110 fs and 1 kHz repetition rate.

## 3 Results and discussion

### 3.1 Morphology and structure analysis

**Figure 1:**
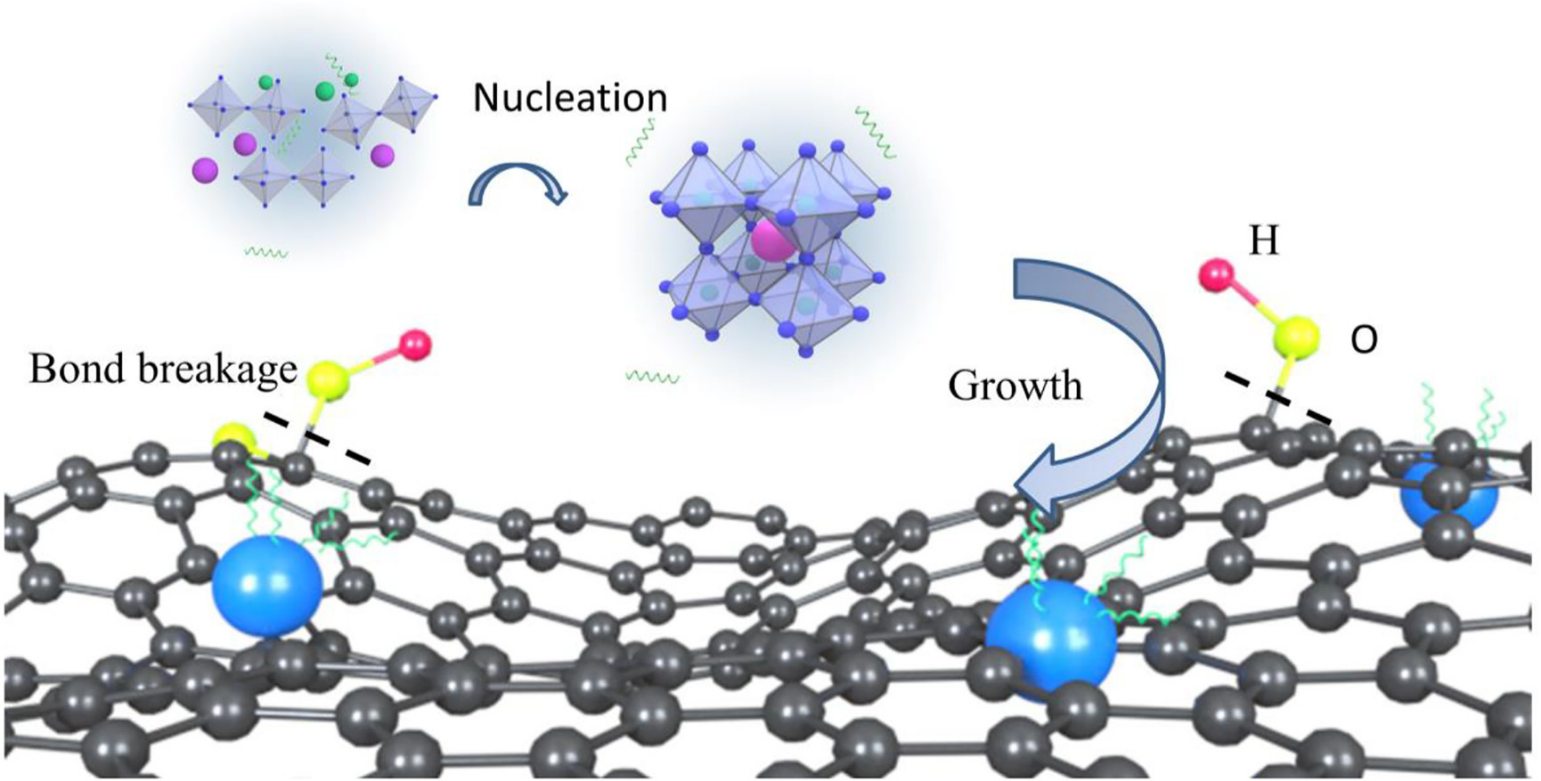
Schematic diagram of CH_3_NH_3_PbBr_3_-G composite nanostructure.

The fabrication schematic diagram of PQDs-G is shown in Figure 1. When DMF coated precursor was injected into toluene solution, high concentration and highly disordered perovskite droplets were formed on the graphene surface, and the oxygen-containing groups of GO were removed during the reaction to form G. The oxygen-containing defect sites on the G surface had high Gibbs free energy, which provided preferential sites for the nucleation and consequently induced the growth of CH_3_NH_3_PbBr_3_ QDs on the G surface.

**Figure 2:**
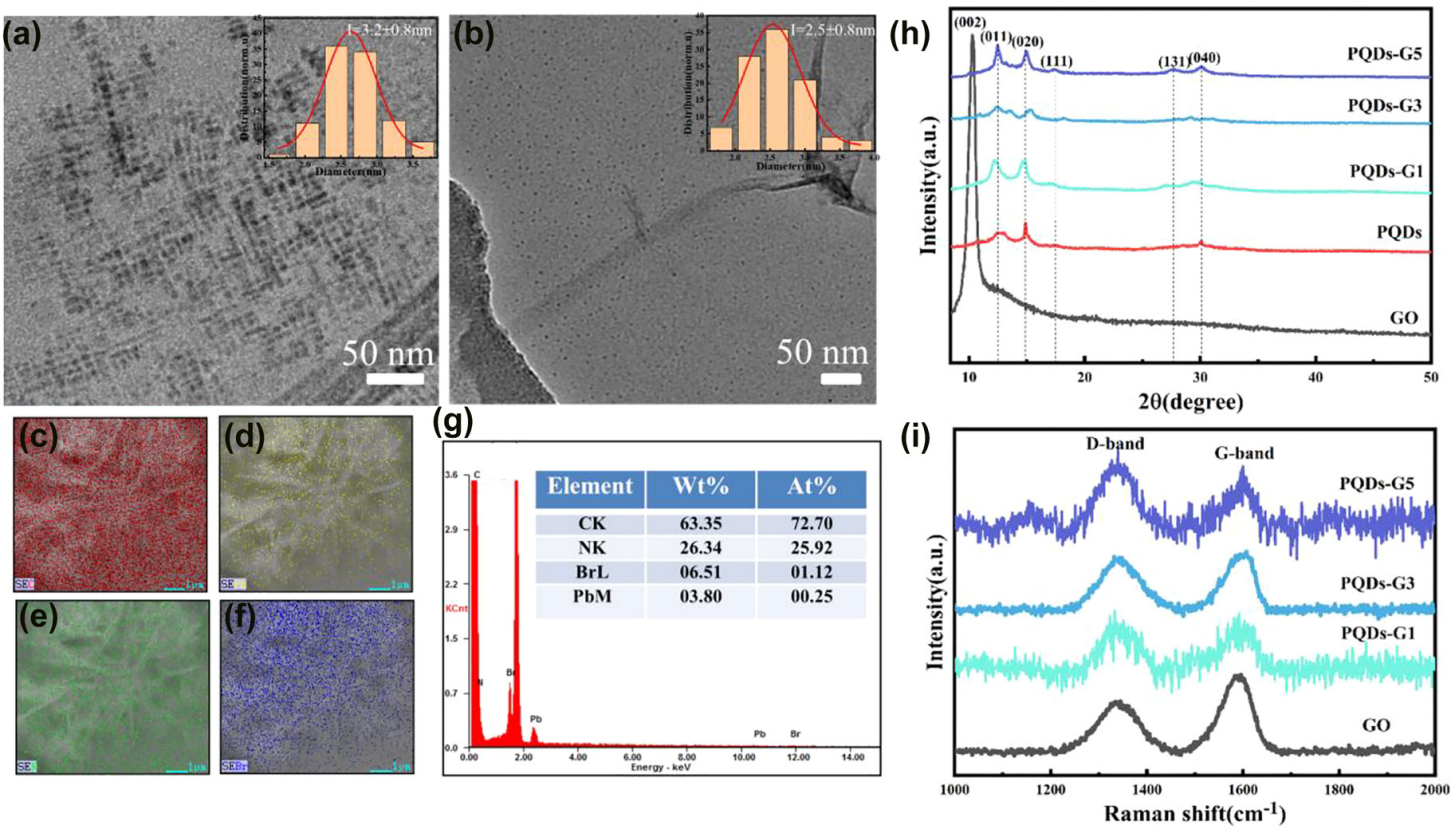
Typical TEM images of (a) PQDs, (b) PQDs-G5. The insets show the size statistics of quantum dots. (c)–(f) The elemental mappings of C, Pb, N, Br in PQDs-G. (g) EDS patterns of G-PQDs. (h) XRD spectra of GO, PQDs and PQDs-G composites. (i) Raman patterns of GO and PQDs-G composites.

The morphologies of the PQDs and PQDs-G5 were characterized via TEM showed in Figure 2(a) and (b). It can be seen that the pure PQDs were synthesized with an estimated average size of 3.2 nm, while the PQDs uniformly distributed on the G nanosheets have an average size of 2.5 nm which is close to the Bohr radius of PQDs [29]. Therefore, the composites may exhibit strong quantum confinement effect. Further morphological characterization shows the PQDs in different composite samples had similar size and distribution (Supplementary Figure S1). As shown in Figure 2(c)–(g), the elemental mappings and EDS of PQDs-G demonstrate that the perovskite in the composites had the same composition as the pure PQDs. The as-synthesized PQDs-G had a Br/Pb molar ratio of 4.48 higher than their stoichiometric ratio which could be attributed to the Br-rich effect of PQDs [30]. Furthermore, it can be seen from the TEM images that the pure PQDs were square-shaped and the PQDs attached to the graphene surface were quasi-spherical. These results could consist with XRD patterns showed in Figure 2(h). A diffraction peak of GO at 10.2° corresponding to the (002) crystalline plane disappeared in the PQDs-G, indicating that GO was reduced to graphene. The main diffraction peaks of PQDs-G and PQDs were located at 2*θ* = 12.47°, 14.86° 17.41°, 27.67° and 30.07°, which were attributed to the (011), (020), (111), (131) and (040) crystal surfaces of CH_3_NH_3_PbBr_3_, respectively. More phase information could be obtained from the changes of the diffraction peaks of the XRD images. The changes of the XRD relative peak intensities on different crystal faces indicate that there was a strong interaction between perovskite and graphene, and the lattice strain was generated in the crystalline perovskite. The introduction of graphene might also affect the stability of PQDs. The pure PQDs and PQDs-G samples exposed in air for 45 days were performed XRD characterization (Supplementary Figure S2). The results show a significant phase change of the pure PQDs while the crystallographic information of the PQDs-G was consistent with the initial. Due to the coupling of G and PQDs, the PQDs could be capped by G to passive the surface trapping states. The excellent chemical stability of G thus improved the stability of composite.

Raman spectra of GO and PQDs-G showed in Figure 2(i) also investigated the interaction between perovskite and graphene. The two prominent features at 1334 and 1589 cm^−1^ in the Raman spectra corresponded to the D band and G band of graphene. The G band came from the stretching of the C–C bond in the plane, and the D band was related to the defect states of graphene [31]. The intensity ratio *I*_D_/*I*_G_ value of PQDs-G increased compared with that of GO, indicating that the surface defects of the composites increased. The interaction between G and PQDs might lead to the fracture and formation of chemical bond.

**Figure 3:**
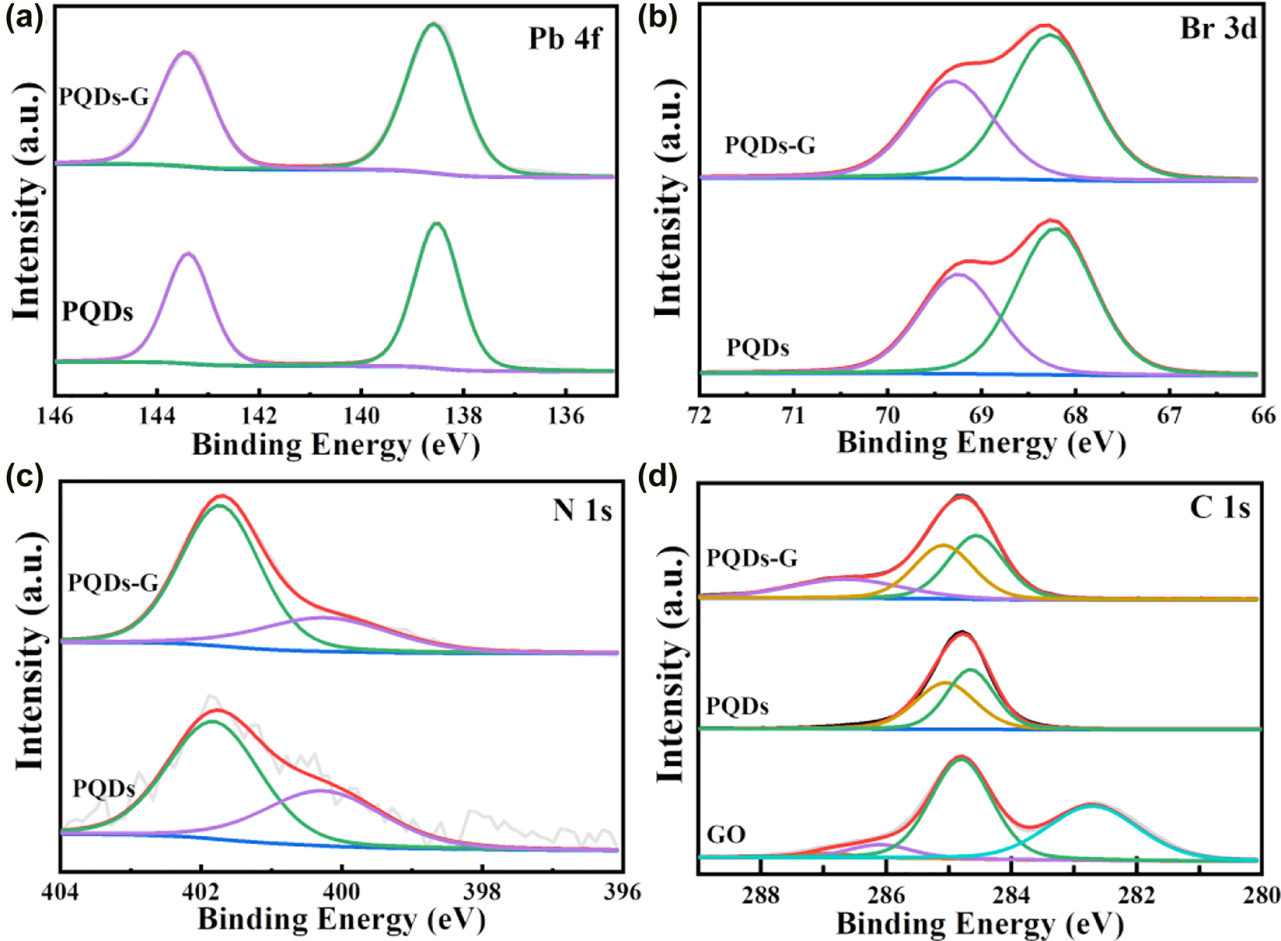
XPS spectrum of PQDs and PQDs-G for the Pb 4f (a), Br 3d (b) and N 1s (c) states. (d) C 1s spectrum of GO, PQDs and PQDs-G.

The XPS were used to demonstrate the channel of the interaction between G and PQDs. As seen in Figure 3, the XPS spectrum of the sample PQDs is basically consistent with the signature peaks of the sample PQDs-G, especially for Pb 4f, Br 3d and N 1s energy states. The Pb 4f spectrum exhibited two peaks positioned at 143.5 and 138.5 eV associated with Pb^2+^ [32]. The Br 3d peaks can be fitted into two peaks with binding energies of 68.2 and 69.3  eV corresponding to the inner and surface ions, respectively [33]. The ratio of intensity of the two bands suggests the existence of Br-rich surface of the samples. The N-1s binding energies confirm the existence of the two chemical environments of the N element with bands at 400.2 and 401.8 eV [33]. These results prove that the PQDs on the G have the same structure and composition as the pure PQDs. In addition, for the C 1s spectrum of PQDs-G, the two peaks with binding energies of 284.6 and 285.1 eV correspond to the C–H/C–C and C–N bonds of PQDs, respectively. However, the spectrum of PQDs-G has an additional characteristic peak at 286.7 eV which is contributed to C–O/C–N bonds compared with the spectrum of PQDs [34]. In order to determine the origin of the additional characteristic peak, the XPS of the GO was also measured and is shown in Figure 3(d) for comparison. The C1s spectrum for GO with peaks at 282.7, 284.82 and 286.12 eV confirm the presence of C–C/C=C, C–C and C–OH bonds [35, 36], respectively. Therefore, we infer that the additional characteristic peak at 286.7 eV in PQDs-G can be attributed to the covalent connection between G and PQDs.

### 3.2 Photophysical properties

The interaction of composite structures could build channel for charge transfer between the different components. Under the laser irradiation, transmission rate and efficiency of the charge transfer process affects the interaction of photons with composite structures, and then affects the photophysical properties of the material.

**Figure 4:**
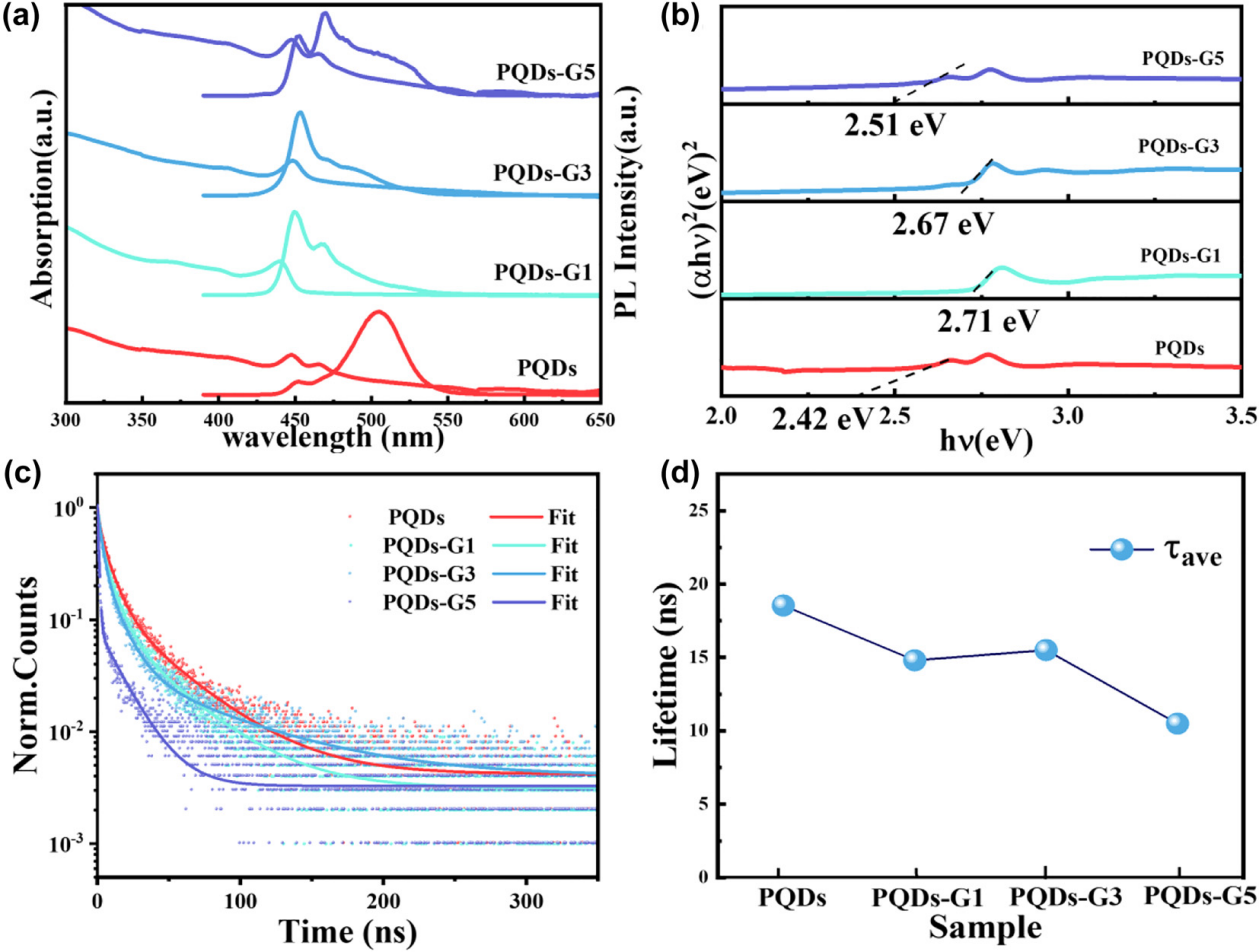
(a) The UV–Vis absorption (left) and PL spectra (right) of PQDs and PQDs-G composites. (b) Tauc plot of all samples. (c) PL decay profiles of all samples. (d) Average fluorescence lifetime of all samples.

Figure 4(a) shows the typical UV–Vis absorption and PL spectra of PQDs and PQDs-G composites. We observed that the absorption peaks of the composites were both relatively blue-shifted compared to that of PQDs. The optical bandgaps of samples were calculated using the Tauc diagram: (*αhν*)^2^ = *A*(*hν* − *E*_g_). The results are shown in Figure 4(b). The blue shift of the absorption spectrum and the increase of the optical band gap directly reflected the change of the optical properties of the composite material. In addition, the composites also have a shoulder peak at around 268 nm, which is related to G (Supplementary Figure S3). GO show an absorption peak at 229 nm coming from the *π*–*π* ^*^ transition of C=C bonds. With the reduction of oxygen-containing group, the restored electronic conjugation would cause the *π*–*π* ^*^ transition redshift to the undisturbed position of G [26]. The PL spectra of composite PQDs-G shows multiple luminescence peaks compared with the pristine PQDs, which we attribute to additional energy transfer pathways provided by graphene. The high electron mobility of graphene changed the excited electronic state of PQDs [37]. Together, these results indicate that the interaction between G and PQDs have changed the optical properties of composites.

To further characterize the photophysical properties of the samples, the time-resolved PL decay curves of the PQDs and PQDs-G samples at the same excitation wavelength of 375 nm were probed, with the corresponding measurement results presented in Figure 4(c). The PL decays were fitted to triexponential decay functions and average PL lifetimes *τ*_ave_ on the nanosecond scale are estimated. The *τ*_ave_ of PQDs was estimated to be 18.54 ns which was similar with previously reported literature of perovskite QDs [29], while the *τ*_ave_ of PQDs-G1, PQDs-G3 and PQDs-G5 are estimated to be 14.81,15.51 and 10.50 ns respectively. We also observed the PLQY of the PQDs-G composites decreased significantly compared with PQDs. The luminescence decay in the composites indicates that the introduction of graphene provided an additional energy-transfer pathway in addition to the intrinsic radiative channel for excited-state electron transfer. Overall, the decrease of PLQY and the accelerated PL decay confirmed that rapid charge transfer occurred between the PQDs and G.

### 3.3 Nonlinear optical properties

The nonlinear optical properties of the prepared samples were investigated using the picosecond Z-scan technique at 532 nm. The samples are in hexane solution with a sample concentration of 0.2 mg/mL and an input pulse intensity of 15 μJ. The nonlinear responses of the solvent irradiated with the same experimental conditions as the samples were found to be very low at 532 nm, which were below the threshold of our detection system, indicating that the obtained Z-scans directly revealed the responses of PQDs and PQDs-G.

**Figure 5:**
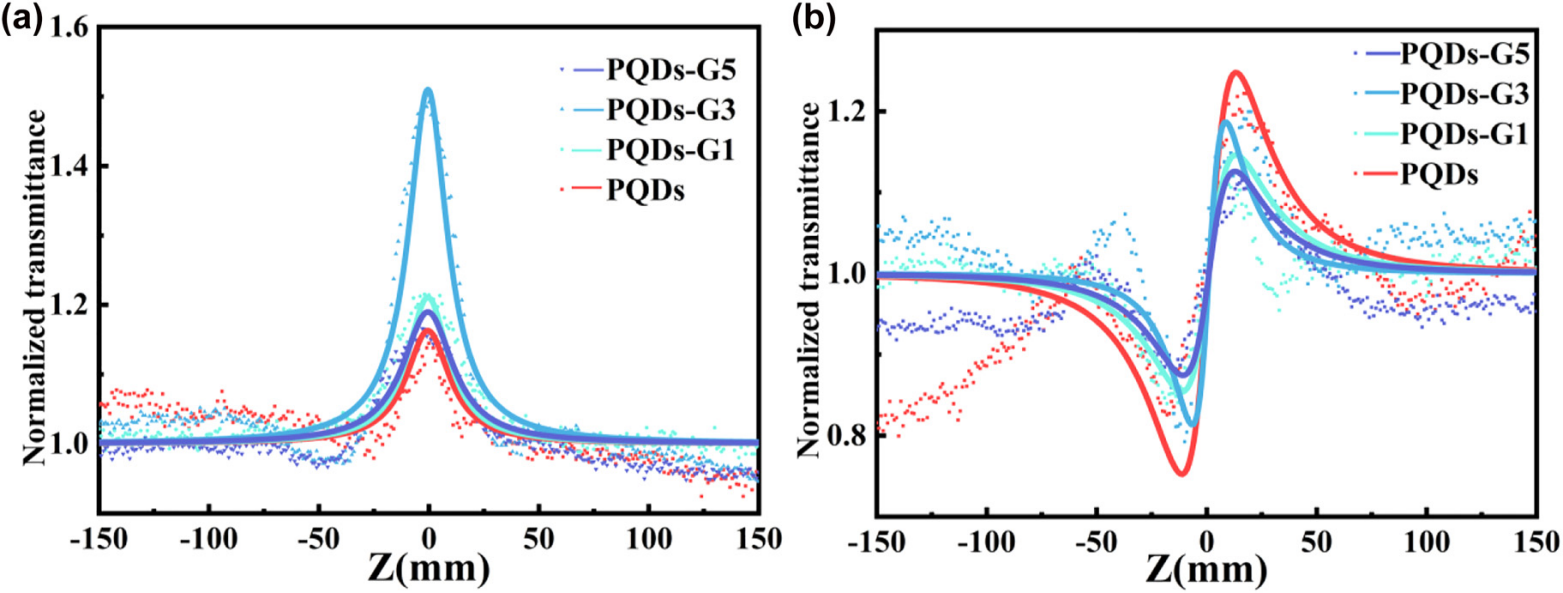
(a) Open aperture Z-scan curves (a) and closed-aperture/open-aperture Z-scan curves (b) of PQDs and PQDs-G composites.

Figure 5(a) gives the open-aperture (OA) Z-scan curves of PQDs and G-PQDs. As the samples move toward the focal point (*z* = 0 mm), the normalized transmissions increase with the increase of the incident intensity, indicating that SA played a dominant role in the nonlinear absorption. The SA can be explained by Pauli-blocking. When the laser irradiated the sample’s atoms, the electrons in the valence band (VB) absorbed the energy of the photons and transitioned to the conduction band (CB). Following Pauli-blocking principle, each electron will occupy an energy state from a low energy state according to the Fermi Dirac distribution. As the incident photon increased, the electron was continuously excited into the CB until the available states in CB have been occupied. The CB can then no longer accept more incoming electrons, which results in the SA. Notably, although the photon energy was slightly smaller than the bandgap energy, the samples still show the SA at 532 nm, which might be explained as the photoexcited electronic transition to the bandgap trap states [13, 38]. Figure 5(b) shows a closed-aperture (CA) Z-scan divided by the open-aperture Z-scan (CA/OA) curves of PQDs and PQDs-G. All the Z-scans resulted in a typical valley-to-peak Z-scan trace, which indicated that all the samples had positive nonlinear refraction and self-focusing properties.

To quantitatively evaluate the NLO properties of all samples, the theoretical fitting curves shown as a solid line in Figure 5(a) and (b) and the third-order nonlinear parameters were obtained using the Z-scan theory. The normalized Z-scan curves of OA were well fitted by the following equation:

(1) $$T\left(z\right)=\overset{\infty }{\underset{m=0}{\sum }}\frac{{\left[-q_{0}\left(z,0\right)\right]}^{m}}{{\left(m+1\right)}^{3/2}}$$

where *T*(*z*) is the OA Z-scan normalized transmittance, *q*_0_(*z*) *=* (*βI*_0_ *L*_eff_)/(1 + *z* ^2^ */z* ^2^_0_), *L*_eff_ = [1 − exp(−*α*_0_ *L*)]/*α*_0_ is the effective length of the sample, *L* is the actual thickness of the sample, *I*_0_ and *z*_0_ are the intensity at the focal point and the diffraction length of the Gaussian beam, respectively. The nonlinear absorption coefficient of the sample can be obtained by the fitting curve: *β* = 2^3/2^(1 − *T*_*z*=0_)(1 + *z* ^2^/*z* ^2^_0_)/*I*_0_ *L*_eff_. The imaginary part of the third-order nonlinear susceptibility is gained by Im*χ* ^(3)^ = *cn* ^2^_0_ *λβ*/480*π* ^3^. As for the nonlinear refraction properties, the normalized CA/OA Z-scan experimental data are fitted by the following equation:

(2) $$T\left(z\right)=1-\frac{4x\Delta \phi_{0}}{\left(x^{2}+9\right)\left(x^{2}+1\right)}$$

Where *x* =  *z/z*_0_, ∆*Φ*_0_ = *k*∆*n*_0_ *L*_eff_ = *kγI*_0_ *L*_eff_ is the on-axis phase shift at the centre focus, *γ* is the nonlinear refractive index coefficient in m^2^/W. The nonlinear refraction index in *esu* is *n*_2_(*esu*) = (*cn*_0_/40*π*)*γ*(m^*2*^/W). Therefore, the real part of the third order nonlinear susceptibility is obtained by Re*χ* ^(3)^ = *n*_0_ *n*_2_/3*π* and the third-order nonlinear susceptibility can be calculated from the formula of *χ* ^(3)^ = [(Re*χ* ^(3)^)^2^ + (Im*χ* ^(3)^)^2^]^1/2^.

Table 1 lists the third-order nonlinear parameters of the samples. It is observed that the Im*χ* ^(3)^ of composites were larger than that of PQDs and the Re*χ* ^(3)^ of composites were smaller than that of PQDs, indicating that the composites enhanced the nonlinear absorption properties and decreased the nonlinear refraction properties of PQDs. Note that, since Im*χ* ^(3)^ is an order of magnitude larger than Re*χ* ^(3)^, the susceptibility *χ* ^(3)^ is almost equal to the Im*χ* ^(3)^ value. Therefore, the enhancement of third-order NLO properties of composites is mainly attributed to the enhancement of nonlinear absorption. The maximum value of *χ* ^(3)^ was 7.09 × 10^−13^ esu, appearing in PQDs-G3, which was approximately 2.6 times that of PQDs.

**Table 1:** The nonlinear susceptibilities of the samples.

Sample	*β*/10^−12^ mW^−1^	Im*χ* ^(3)^/10^−13^ esu	Re*χ* ^(3)^/10^−14^ esu	*χ* ^(3)^/10^−13^ esu
PQDs	−5.11	−2.19	16.49	2.74
PQDs-G1	−7.02	−3.01	9.76	3.16
PQDs-G3	−16.28	−6.98	12.48	7.09
PQDs-G5	−6.06	−2.60	8.21	2.73

**Figure 6:**
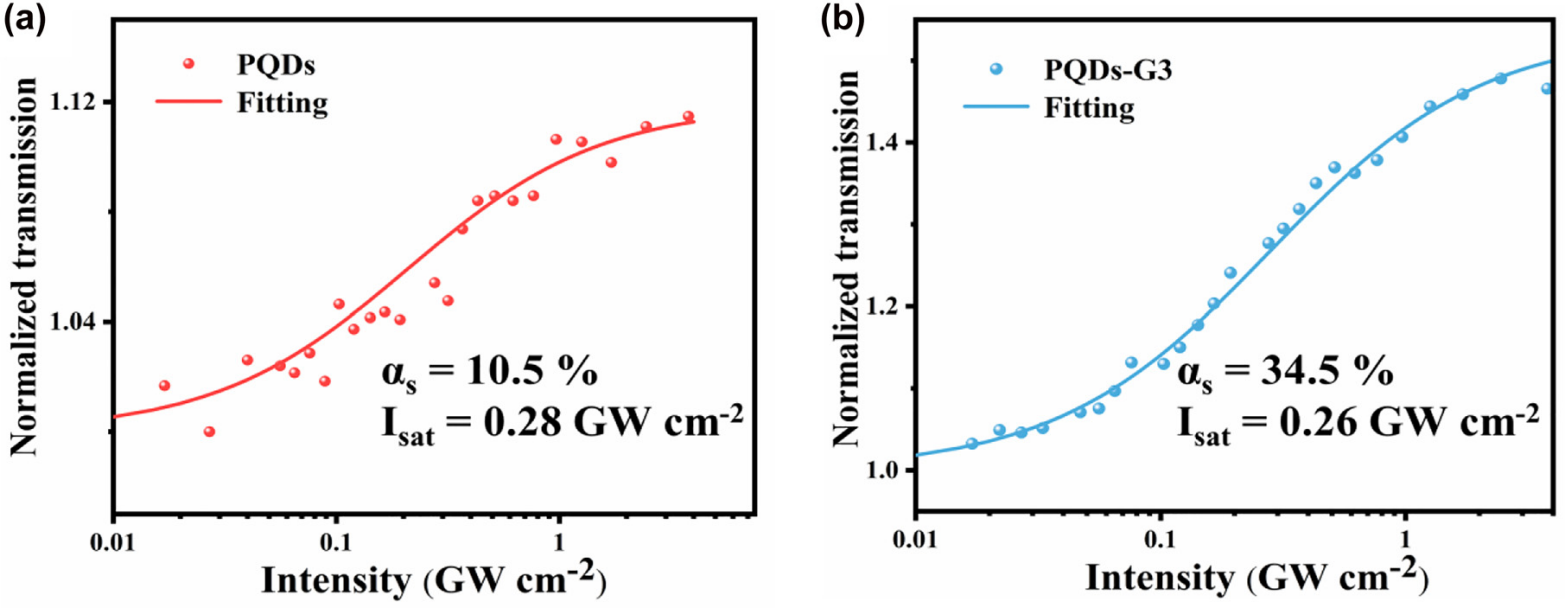
Normalized transmission versus input intensity of PQDs (a) and PQDs-G3 (b).

To further evaluate the nonlinear absorption properties of samples, we recorded the normalized transmission versus input intensity of PQDs and PQDs-G3 in Figure 6(a) and (b). The obtained data can be fitting by the formula:

(3) $$T=1-\frac{\alpha_{\text{s}}}{1+I/I_{\text{sat}}}-\alpha_{\text{ns}}$$

where *α*_ns_ is the non-saturable component, *α*_s_ is the modulation depth, *I*_sat_ is the saturable intensity.

We obtained that the α_s_ of 10.5% and 34.5% for the PQDs and PQDs-G3 corresponded to the *I*_sat_ of 0.28 GW cm^−2^ and 0.26 GW cm^−2^. The *I*_sat_ of PQDs-G is similar to that of PQDs, whereas *α*_s_ of PQDs-G is bigger. For evaluating the saturable absorption properties of PQDs-G composite, we performed comparisons to the nonlinear absorption parameters in different SA materials, as shown in Table 2. The modulation depth is the maximum change of transmittance when pulsed into the saturation absorber, and a high modulation depth implies the composite has strong ground-state bleaching ability for incident light, which is more beneficial for the saturable absorption effect. The large saturable absorption intensity and low saturable absorption coefficient are the biggest obstacles restricting graphene in laser pulse shaping devices. We found that the PQDs-G exhibited a high nonlinear absorption coefficient and a low saturation intensity, indicating that the PQDs-G can easily achieve SA under picosecond light excitation. These results directly suggest that the PQDs-G composite may find important applications in mode-locked laser and Q-switching for ultrafast pulsed lasers.

**Table 2:** Nonlinear absorption parameters of different materials.

Sample	*λ* (nm)	*β* (cm/GW)	*α*_s_ (%)	*I*_sat_ (GW/cm^2^)	Reference
PQDs	532	−0.511	10.5	0.28	This work
PQDs-G	532	−1.628	34.5	0.26	This work
G	532	−0.012	–	265	[39]
CsPbBr_3_	515	−0.350	–	11	[40]
MoSe_2_	800	−0.044	15.1	0.23	[41]
Ag-BPNF	532	−1.01	19.8	18.6	[42]

### 3.4 Mechanistic understanding of the enhanced NLO properties

The enhanced NLO properties may be related to the change of the individual components of PQDs and the synergistic effect of the two components between the composite structures. The photoinduced transition dipole moment generated by the exciton oscillator strength was considered a significant factor affecting the NLO performance of QDs. Normally, the electrons were localized by the QDs size, and the wave functions of the electron and the hole in the two-pair exciton became increasingly overlapped with size decrease, which enhanced the oscillation intensity and susceptibility. While in the strong quantum confinement region, the exciton strength decreased with size further decrease due to the coulomb repulsion force, which limited the NLO properties [43]. According to the TEM images, PQDs of composites are strongly quantum confined and their size is close to that of pure PQDs. Therefore, the size of PQDs had little effect on the enhanced NLO properties of the composite.

The characterization of photophysical properties in this research has shown the existence of ultrafast charge transfer between G and PQDs which is strongly relevant to the saturable absorption. In order to understand the mechanism of the observed enhanced saturable absorption of PQDs-G composite, we carried out femtosecond transient absorption (TA) spectroscopy characterizations to track the ultrafast carrier dynamics in the samples. The TA spectra were recorded by exciting at 380 nm (above band edge) and probing with a light (425–560 nm) as a function of time delay between pump and probe pulses.

**Figure 7:**
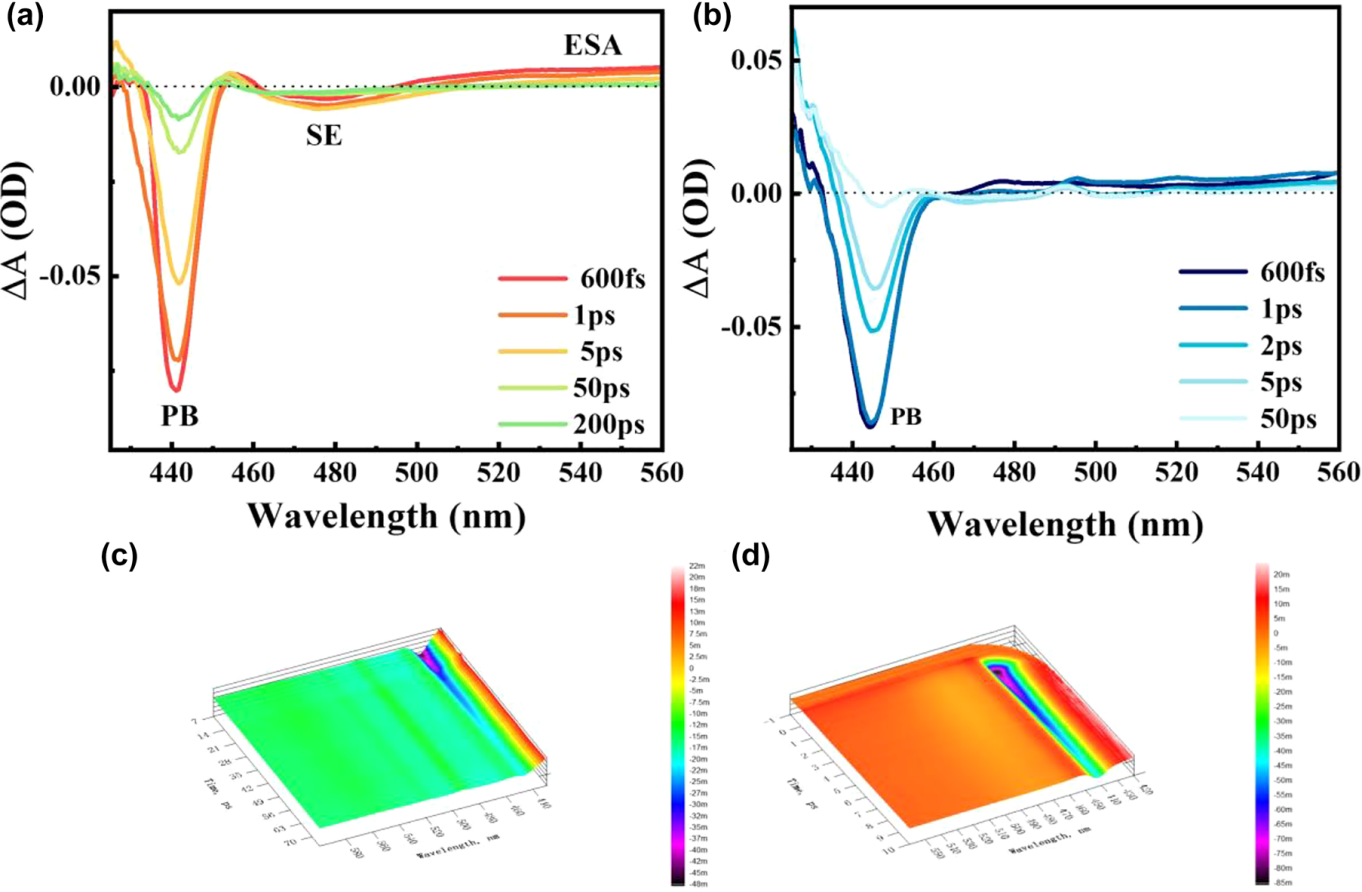
2D transient absorption (TA) spectra of PQDs (a) and PQDs-G (b) at different delay time, with the excitation at 380 nm. 3D TA spectra of PQDs (c) and PQDs-G (d).

Figure 7 shows 2D and 3D transient absorption spectra of PQDs and PQDs-G at several delay times. The TA spectra of PQDs show two negative signals in the short wavelength region, which may be attributed to photobleaching (PB) or stimulated emission (SE). Since the centres of PB and SE are expected to match those of the band edge absorption peaks and PL peaks, respectively, the negative signal at 480 nm is attributed to SE and the negative signal at 445 nm is attributed to PB [44, 45]. The long wavelength region is dominated by a broad positive (498–560 nm) corresponding to photoinduced excited state absorption (ESA). The strong excited state of PQDs will weaken the intensity of saturated absorption. For the TA curve of PQDs-G, we only observed a similar PB signal at 440 nm and a weak ESA signal compared with PQDs. These results indicate that the addition of G changed the excited electronic state of PQDs, which weaken the ESA and enhanced the saturated absorption.

Comparing the evolution of negative signals of PQDs and PQDs-G can better analyse the reasons for the enhancement of saturated absorption. The PB signals were attributed to the depletion of ground state electrons and filling of excited states. Photoexcitation induces the generation hot electrons in the conduction band and hot holes in the valence band. After photoexcitation electrons and holes thermalize to the quasi-equilibrium distributions, the nonlinear absorption reaches saturation with a high carrier density. The time scale of this process is usually shorter than 1 ps. Therefore, by observing the PB signals at 600 fs, we obtained that the value of absorption intensity of PQDs is −0.08 and the value of absorption intensity of PQDs-G is −0.088, indicating that the carrier density in the composites was higher than that of pure PQDs. In addition, the presence of SE signal is due to the carrier density of PQDs reaching the threshold. Therefore, the disappearance of the SE signal in PQDs-G indicates the carrier density threshold is higher for PQDs-G than PQDs. The higher carrier density and threshold indicate the stronger saturated absorption capacity of PQDs-G. The recovery of PB signal indicates the recombination process of photogenerated electrons and holes, and the time-resolved TA spectra give initial evidence that the PQDs-G had a faster carrier recombination rate. Considering the fact that intraband thermal exciton relaxation (relaxation of hot excitons to the band-edge excitonic state) might also happen [46], therefore we performed a normalized kinetic fit to further understand the ultrafast kinetic of PQDs-G and PQDs excited state and the charge transfer process.

**Figure 8:**
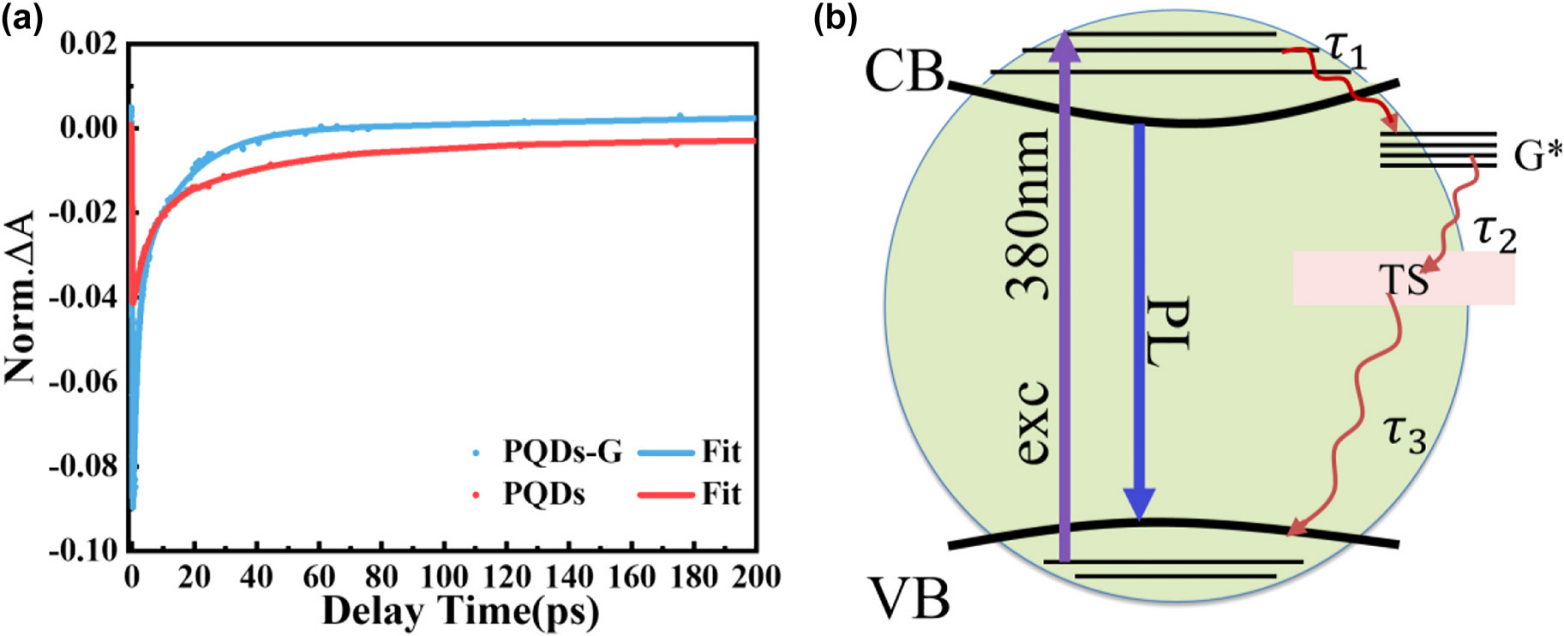
(a) TA decay kinetics (dotted lines) with triple exponential fitting curves (solid line) of PQDs (red) and PQDs-G (blue) under 380 nm excitation. (b) Schematic representation of the proposed charge transfer model in PQDs-G.

Figure 8(a) shows the normalized kinetic decay profiles. The decay profiles were fitted with a triple exponential function (solid line), and the corresponding fitting parameters are reported in Table 3. The resulting characteristic time parameters are as follows: *τ*_1_ = 5.170 ps, *τ*_2_ = 38.39 ps, *τ*_3_ = 539.2 ps for the PQDs, while *τ*_1_ = 5.170 ps, *τ*_2_ = 38.39 ps, *τ*_3_ = 539.2 ps for the PQDs-G. The carrier relaxation time of PQDs was accelerated by the regulation of the composite structure, which was consistent with the PL lifetime results. The *τ*_1_, *τ*_2_ and *τ*_3_ components correspond to the fast, medium and slow decay processes for each sample. Previous research has explained the dynamics of exciton decay in perovskite [47]. The fast decay is attributed to the intraband thermal exciton relaxation. The medium decay is attributed to the exciton trapping to the bandgap trap states. The slow decay of near 500 ps can be attributed to the nonradiative recombination process of carriers. The accelerated fast decay and medium decay can be understood as the result of charge transfer between PQDs and G. The charge transfer process will introduce new energy levels, leading to increased density of the lowest excitonic state. The increase of density of states would promote the coupling between the high exciton state and the lowest exciton and the coupling between the lowest exciton state and the trap states (TS) corresponding to the *τ*_1_ and *τ*_2_ processes, respectively. This argument is consistent with the analysis of the TA spectrum.

**Table 3:** Fitting parameters of TA Decay of PQDs and PQDs-G fitted with triple exponential function.

Sample	*τ*_1_(ps)	*τ*_2_(ps)	*τ*_3_(ps)
PQDs-G	1.497	14.62	466.2
PQDs	5.170	38.39	539.2

The acceleration of slow decay process of PQDs-G and the results of PLQY can help to analyse the direction of charge transfer. PQDs shows a high PLQY due to its carrier recombination process carried out in a nonradiative form. The fluorescence quenching of PQDs-G indicates that the emission channels of the excited electrons changed. Figure 8(b) shows the charge transfer model of PQDs-G. Upon the irradiation of a laser pulse, the electrons in the VB of PQDs rapidly transitioned to the CB and from the excited state to a higher excited state. Due to the high carrier mobility of G, more excited electrons were transferred to G instead of returning to VB of PQDs in the form of fluorescence. The ultrafast charge transfer process changed the electronic state of the composite structure, leading to remodulation of the absorption cross section and thus enhanced the saturated absorption properties of PQDs-G.

The analysis in this section shows that the charge transfer process provides an energy transfer channel between PQDs and G to obtain stronger NLO properties, compensating for the limited regulation of individual PQDs component. In addition, another unique advantage of the charge transfer process is that it can effectively tune the carrier dynamics in the composites to obtain improved nonlinear absorption properties. The ultrafast carrier dynamics process also indicates that the carrier relaxation time of PQDs could be tuned by G and the PQDs-G composite integrated the excellent carrier transport properties of graphene, which is suitable for fast-response nonlinear optical devices.

## 4 Conclusions

In this study, we have successfully synthesized PQDs-G nanohybrids by growing CH_3_NH_3_PbBr_3_ QDs from graphene oxide by a defect-mediated crystal growth technique. We found that the PQDs-G integrated the advantages of G and PQDs due to the charge transfer process. Picosecond Z-scan measurements indicated the PQDs-G composite exhibited improved saturable absorption properties with large modulation depth (34.5%) and low saturation intensity (0.26 GW cm^−2^). By controlling the content of the G, the saturation absorption coefficient *β* of PQDs-G was 3.19 times larger than that of PQDs. Using the transient absorption experimental data and kinetic modelling, we studied the carrier dynamics of the samples and correlated it with the nonlinear absorption process. The results show that the ultrafast charge transfer in the composite enhanced the exciton coupling, leading to the enhancement of saturation absorption effect. The PQDs-G composite presents a potential candidate as saturable absorber of passive mode-locking or Q-switching device for ultrafast pulsed lasers.

## Supplementary Material

Supplementary Material Details
