# Supplementary material for: Ultrafast charge transfer enhanced nonlinear optical properties of CH3NH3PbBr3 perovskite quantum dots grown from graphene
Source: Nanophotonics. 2022 May 27;11(13):3177–88. doi: 10.1515/nanoph-2022-0251 (PMC11501371; doi:10.1515/nanoph-2022-0251)
Supplement: Supplementary file 1 — Supplementary Material Details [file j_nanoph-2022-0251_suppl.doc]

**Supplementary Material** for

**Ultrafast charge transfer enhanced nonlinear optical properties of CH_3_NH_3_PbBr_3_ perovskite quantum dots grown from graphene**

*Ye Yuan^#^, Fenglin Cao^#^,* *Peng Li, Jiawen Wu,* [*Baohua*](#_bookmark0) *Zhu^*^ and Yuzong Gu^*^*

*Physics Research Center for Two-Dimensional Optoelectronic Materials and Devices, School of Physics and Electronics, Henan University, Kaifeng 475004, China*


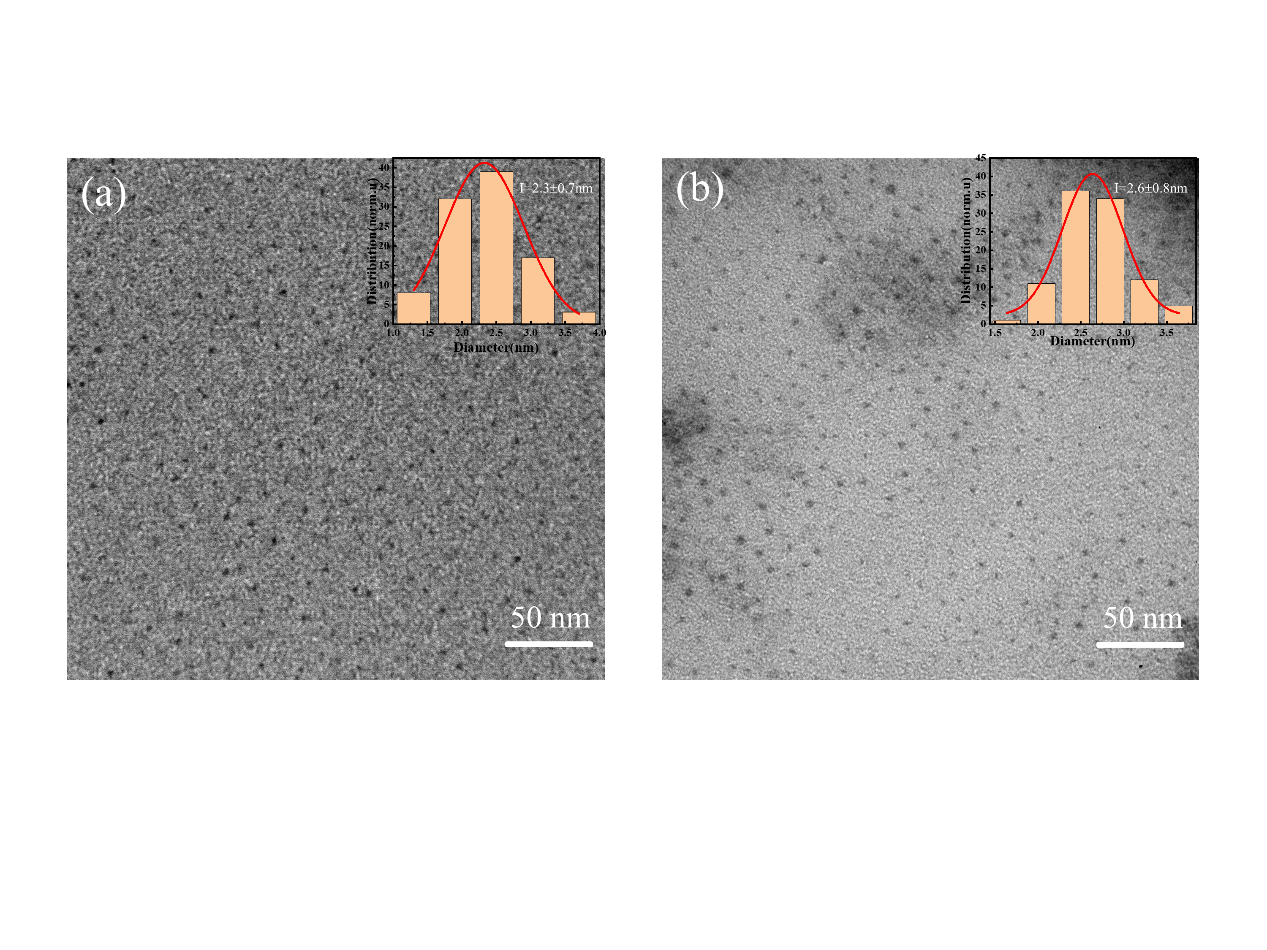


**Figure S1: The TEM images of (a) PQDs-G1, (b) PQDs-G3. The insets show the size statistics of PQDs grown on G.** PQDs in different PQDs-G composite samples have similar distribution and size. The PQDs of PQDs-G1 have an average diameter of 2.3 nm with a size deviation of 0.7 nm, and PQDs of PQDs-G3 have an average diameter of 2.6 nm with a size deviation of 0.8 nm.


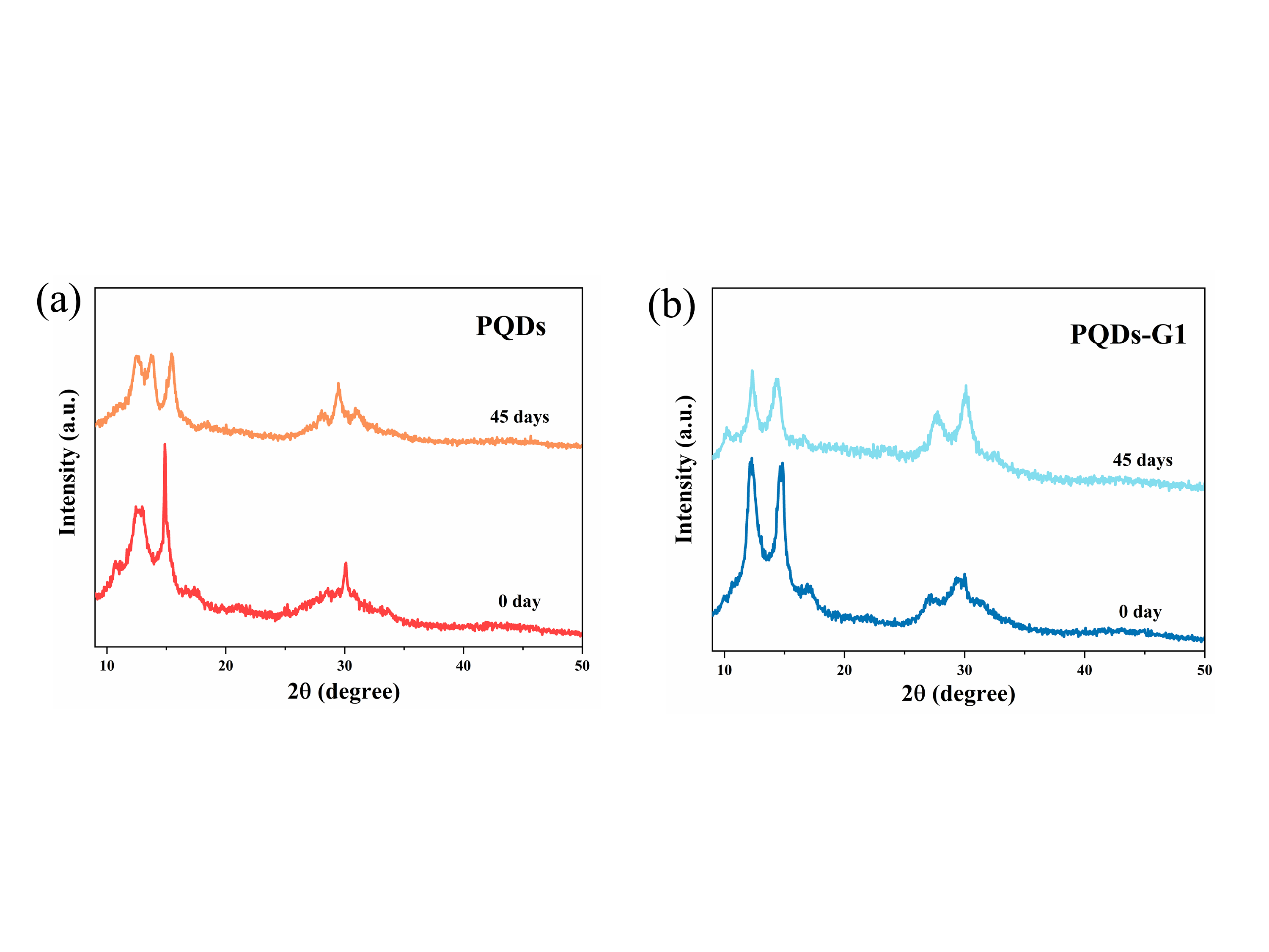


**Figure S2: The time-evolution of the XRD patterns of (a) pure PQDs, (b) PQDs-G1 exposed to the air for 0 day and 45 days.** XRD was used to investigate the changes in the crystallinity and phase purity of pure PQDs and PQDs-G composite. The XRD diffraction peak around 12° split into two peaks after PQDs was exposed to the air for 45 days while the XRD diffraction peak of the PQDs-G composite had no obvious change, which indicate the stability was improved by the introduction of G.


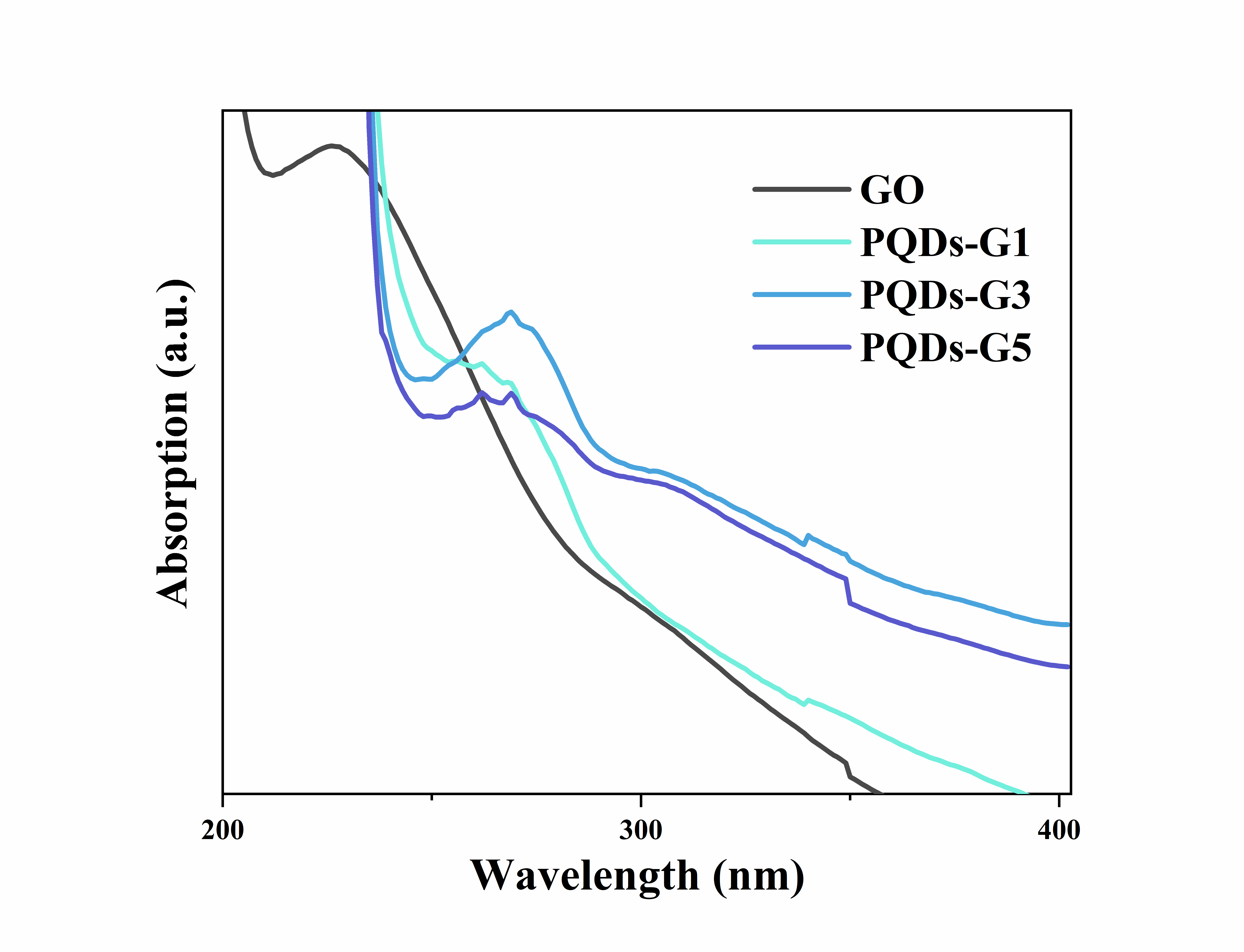


**Figure S3: The UV-Vis absorption spectra of GO and PQDs-G in the range of 200-400 nm.**
